# First evidence of terrestrial ambrein formation in human adipocere

**DOI:** 10.1038/s41598-019-54730-w

**Published:** 2019-12-04

**Authors:** Barbara von der Lühe, Robert W. Mayes, Volker Thiel, Lorna A. Dawson, Matthias Graw, Steven J. Rowland, Sabine Fiedler

**Affiliations:** 1 Institute of Geography, University of Mainz, Johann-Joachim-Becher-Weg 21, 55099 Mainz, Germany; 2 The James Hutton Institute, Craigiebuckler, Aberdeen, AB15 8QH Scotland UK; 3 Geobiology, Geoscience Centre, University of Göttingen, Goldschmidtstraße 3, 37077 Göttingen, Germany; 4 Institute of Forensic Medicine, University of Munich, Nußbaumstraße 26, 80336 Munich, Germany; 5 Petroleum and Environmental Geochemistry Group, Biogeochemistry Research Centre, University of Plymouth, Drake Circus, Plymouth, PLA 8 AA UK; 6 Present Address: Physical Geography, Institute of Geography, University of Göttingen, Goldschmidtstraße 5, 37077 Göttingen, Germany

**Keywords:** Biogeochemistry, Sterols, Geochemistry

## Abstract

To date, the only known occurrence of ambrein, an important perfumery organic molecule, is in coproliths found in about one in a hundred sperm whales. Jetsam ambergris coproliths from the whale are also found occasionally on beaches worldwide. Here we report on the surprising occurrence of ambrein in human adipocere. Adipocere is a waxy substance formed post-mortem during incomplete anaerobic decomposition of soft tissues. Adipocere samples obtained from grave exhumations were analysed using gas chromatography-mass spectrometry (GC-MS). In addition to the typical fatty acids of adipocere, lesser amounts of ambrein were identified in the samples, in abundances similar to those of the major accompanying faecal steroids. The distribution of these compounds suggests that ambrein was produced post-mortem during the microbial decomposition of faecal residues and tissues. It is assumed that the adipocere matrix of saturated fatty acidsaided the preservation of ambrein over extended periods of time, because adipocere is stable against degradation. The association of ambrein formation in ageing faecal material, under moist, oxygen-depleted conditions, now requires more attention in studies of other mammalian and geological samples. Indeed, ambrein and its transformation products may be useful novel chemical indicators of aged faecal matter and decomposed bodies.

## Introduction

**Figure 1:**
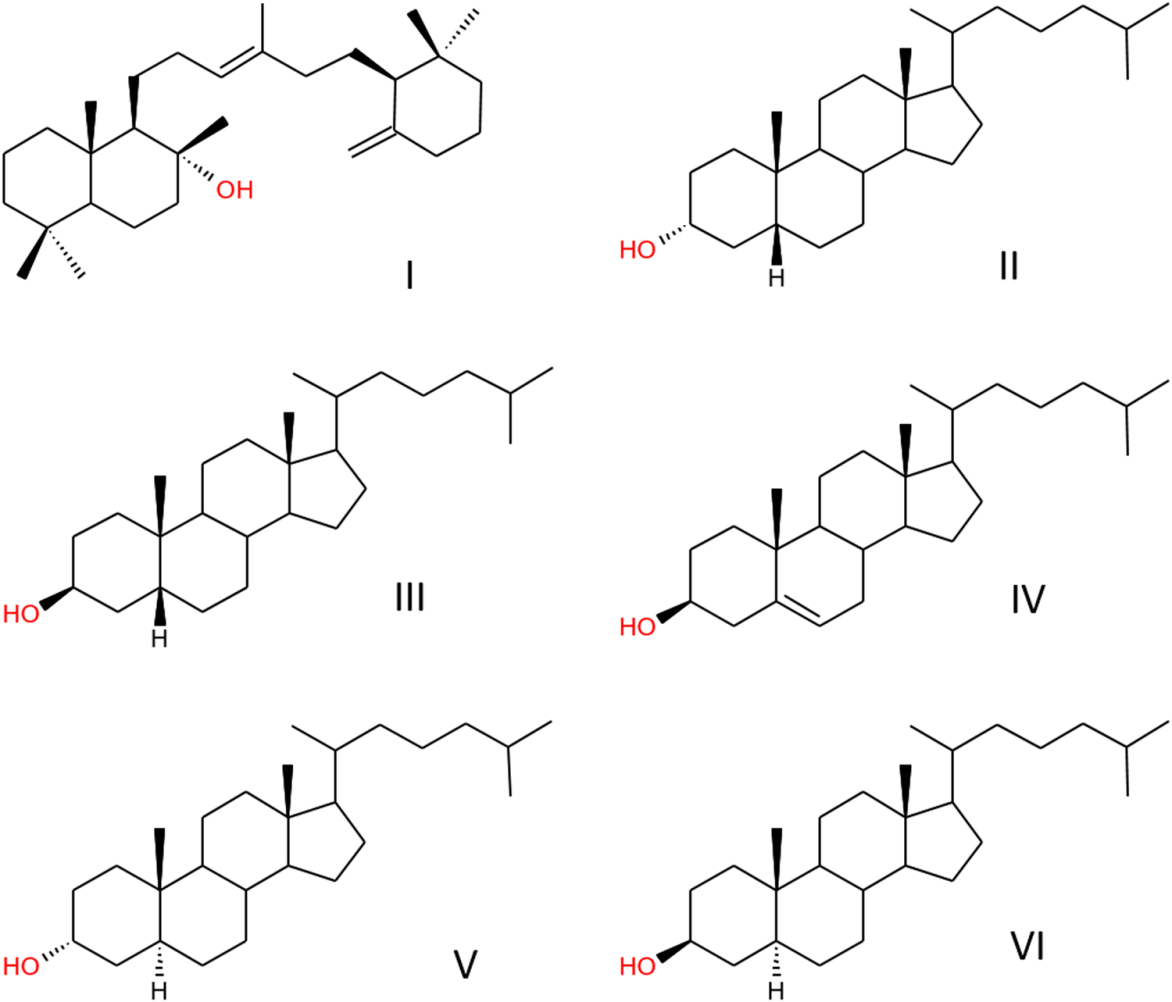
Structures of compounds discussed in the text. (I) ambrein; (II) epicoprostanol; (III) coprostanol; (IV) cholesterol; (V) epicholestanol; (VI) 5α-cholestanol.

The tricyclic triterpenoid, ambrein (**I**, Fig. 1), a perfumery chemical of long historical importance is, to date, only known in the natural world as the major component of ambergris, a coprolith originating from about 1% of sperm whales (*Physeter macrocephalus*)^1,2^. In the sperm whale, coprolith formation is associated with the indigestible horny beaks of squid, their principal food, which may occasionally pass through the intestines and form a faecal concretion in the rectum^2^. After excretion into the environment, ambergris coproliths float in the ocean and after being washed ashore, are sometimes collected on beaches^3^. Such ‘jetsam’ ambergris is a rare and valuable odorant and perfume fixative that has been fascinating mankind for centuries. The weathering and ageing process of floating ambergris enhances its quality for perfumery usage, as the scentless ambrein is oxidized (autoxidation or photo-oxidation) to various odorous products^2,4,5^.

Surprisingly, little is known about the chemical composition of ambergris and the formation of ambrein in the coproliths. Studies were mainly conducted in the early 20^th^ century^1,4,6^ but recently, new insights into the identification of jetsam ambergris have been made^3,7,8^. These have shown that, in addition to ambrein, steroids including epicoprostanol (**II**), coprostanol (**III**) and coprostanone are typical components of ambergris^1,3,9^. These compounds can be regarded as microbial transformation products derived from cholesterol (**IV**), which reflect the origin of ambergris in the rectum, where the masses occur alongside liquid faecal matter^2^. Other minor constituents in ambergris are ambrein derivatives and minor amounts of fatty acids including palmitic, stearic, oleic, linoleic, arachidonic and behenic acids^5,10,11^. The odorous volatile oxidation products of grey ambrein in fact only account for a small proportion of ambergris (<0.5% by mass)^12,13^. Whereas most of the literature is focused on the use of ambrein in the perfumery industry, particularly on artificial ambrein synthesis and the artificial production of its odorous derivatives^10,12,14–16^, the processes controlling its natural biosynthesis associated with ambergris are largely unknown. It has been suggested that ambrein is an intestinal microbial transformation product, possibly derived from partial cyclisation of squalene: indeed this has been demonstrated *in vitro*^2,17^. Likewise, precursor molecules of ambrein formation (e.g. 3-deoxyachilleol) have been identified *in vitro*^2,17,18^, but it is not known yet whether these molecules are also responsible for ambrein formation in a natural environment^2,17,18^.

No natural sources of ambrein, other than whales, have been discovered so far. Here, we report on the discovery of ambrein in human adipocere, which is a post-mortem decomposition product of soft fatty tissues. We thus demonstrate, for the first time, that ambrein can also be formed in terrestrial settings. Likely, ambrein is a microbial transformation product, linked to similar biochemical precursors, microorganisms and environmental conditions in both marine and terrestrial settings.

## Ambrein and Steroids in Human Adipocere

**Figure 2:**
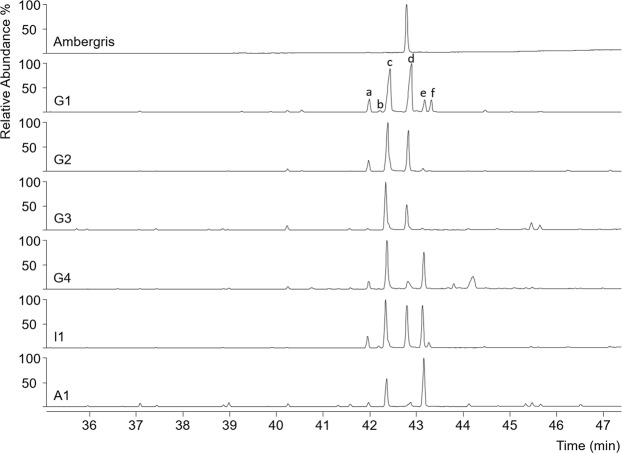
Partial (35 min to 47 min) total ion current GC-MS chromatograms of silylated extracts of ambergris, neutral lipids of adipocere from graves (G1–G4), neutral lipids of intestinal content from adipocere sample G2 (I1) and neutral lipids of external adipocere (A1). Components (as TMS ethers): a = coprostanol; b = epicholestanol; c = epicoprostanol; d = ambrein; e = cholesterol and f = 5α-cholestanol. The ambergris extract was diluted 1:100 v/v compared to adipocere samples (G1–4; A1) and the intestinal sample (I1). Note that the major lipids of adipocere (fatty acids) are not included in the analyses.

We analysed the neutral lipids of human adipocere samples from four grave exhumations from a cemetery in South West Germany (G1–4) and from the intestinal content of G2 (I1) (Table 1). The adipocere samples were obtained after an *in situ* burial of ~40 years. Further, one external adipocere sample was analysed (A1), as a reference to demonstrate that ambrein formation is not specific for the grave exhumations (Table 1). This reference sample derives from an adipocere corpse that has been submerged under water (Table 1). Preliminary observations on the neutral lipid composition of 40 adipocere specimens from the cemetery in SW Germany were carried out and GC-MS analysis revealed that 10 of these samples contained a compound that had, according to the NIST 05 library, a mass spectrum similar to ambrein. Four samples were selected with the additional external adipocere sample and were subsequently investigated in more detail. The GC-MS results were compared to those obtained for a sample of jetsam ambergris, using the same method of analysis, and with published data^3,19^. As expected, GC-MS chromatograms of derivatised neutral lipids (Fig. 2) showed ambrein (as the trimethylsilyl (TMS) ether derivative) as the major component in jetsam ambergris. Ambrein also occurred, in lower relative abundances, in the adipocere samples (i.e. the grave adipocere (G1–G4)), intestinal content of G2 (I1) and external adipocere sample (A1) (Fig. 2 and Table 2). Further abundant compounds observed in the adipocere were coprostanol, epicholestanol (**V**), epicoprostanol, cholesterol and 5α-cholestanol (**VI**) (Fig. 1). The electron ionization (EI) mass spectrum of ambrein (−TMS ether) from the adipocere sample G2 was virtually identical to that of derivatised ambrein from the authentic ambergris sample (Fig. S1, Supplementary Material; M^+^ at *m/z* 500, M^+^-CH_3_ at *m/z* 485, base fragment ion at *m/z* 143^3,19^). The identification of ambrein in the adipocere was further confirmed by co-elution experiments with ambrein in the authentic ambergris sample and sample G2 (Fig. S2, Supplementary Material). This also revealed a retention index of 3095 for the TMS ether of ambrein on a ZB-5ms stationary phase (Phenomenex, Torrance, USA), which was slightly different to that previously reported for a HP-5ms stationary phase (retention index 3110)^3^. Between the adipocere samples studied, the relative abundances with respect to the summed co-occurring neutral steroids and fatty acids varied considerably (Table 2). Ambrein was most abundant in G1 (2.95%), G2 (1.37%) and in the intestinal sample of G2 (I1, 1.25%; Table 2). Lower abundances were detected in G3 (0.04%), G4 (0.03%) and in the external adipocere sample (A1; 0.02%, Table 2), where the identification of ambrein was additionally hampered by near co-elution with the major amounts of *n*-hentriacontane. Among the steroids, epicoprostanol was most abundant in all samples, including ambergris (Fig. 2 and Table 2). Its precursor, cholesterol, was present in low amounts in G2 and G3 adipocere and in higher abundances in G4, I1 and in the external adipocere sample (A1). Coprostanol was a minor compound in all adipocere samples and in the intestinal sample of G2 (I1). G1 and I1 also had low relative concentrations of epicholestanol (Fig. 2).

**Table 1:** Adipocere samples analysed in this study.

Sample	Age at burial	Sex	Burial/submergence duration (years)	Weight of human remains (kg)
G1	81	f	39	31.5
G2, I1	86	m	39	20
G3	24	m	36	10.5
G4	30	m	36	23
A1	88	f	n.k.	n.k.

G1–G4 are samples from separate graves in a cemetery located in SW Germany; I1 = intestinal contents sample from G2; A1 = external adipocere sample from the submerged body.

f = female, m = male, n.k. = not known.

**Table 2:** Concentrations (mg g^−1^) of steroids (sterols as TMS ethers), ambrein (as TMS ether) and dichloromethane soluble (free) fatty acids (as methyl esters) in adipocere samples from grave exhumations (G1–4), the intestinal content of G1 (I1), the external adipocere sample (A1) and in ambergris.

Sample	Compound group	G1	G2	G3	G4	I1	A1	Ambergris
**Concentrations [mg g**^**−1**^**]**								
Coprostanol	Isoprenoids	0.7	0.2	0.03	0.1	0.5	0.1	17.7
Epicholestanol		0.1	n.d.	n.d.	n.d.	0.1	n.d.	n.d.
Epicoprostanol		3.9	1.7	0.9	0.5	2.8	0.9	20.3
Ambrein		4.2	1.1	0.3	0.1	2.3	0.2	662.9
Cholesterol		0.2	0.1	0.1	0.3	2.1	0.9	n.d.
5α-Cholestanol		0.3	0.01	n.d.	0.007	0.3	0.01	n.d.
C_14:0_	FAME	2.8	1.0	94.5	26.4	4.9	99.5	n.d.
C_16:0_		89.6	57.8	704.3	388.9	144.2	704.6	2.8
C_18:2_		n.d.	n.d.	n.d.	10.1	n.d.	4.8	n.d.
C_18:1_		3.5	3.6	8.1	14.3	8.3	31.7	n.d.
C_18:0_		26.4	16.3	82.7	37.2	19.4	99.9	n.d.
10-OH-C_18:0_		11.9	0.4	3.1	3.4	0.8	18.3	n.d.
C_20:0_		n.d.	n.d.	n.d.	n.d.	n.d.	n.d.	0.6
C_22:0_		n.d.	n.d.	n.d.	n.d.	n.d.	n.d.	1.3
**Total extractable lipids**		**143**.**7**	**82**.**3**	**894**.**0**	**481**.**5**	**185**.**9**	**960**.**9**	**705**.**7**
**Relative concentrations [%]**								
Ambrein	Relative to total lipids	2.95	1.37	0.04	0.03	1.25	0.02	93.9
FAME		93.4	96.2	99.9	99.8	95.6	99.8	0.7

Concentrations were determined with internal standards (IS; steroids = 5α-cholestane, fatty acid methyl esters = C_19:0_) added prior to the GC-MS analysis. Relative concentrations (%) of ambrein and FAME are expressed as being relative to the total abundances of steroids and FAME (=total lipids) in adipocere and ambergris samples.

n.d. = not detected; FAME = fatty acid methyl ester.

## Constituents of Adipocere and Their Relation To Ambrein

Adipocere is a product of limited human tissue decomposition and is typically associated with anaerobic, moist and oxygen-depleted environments^20,21^. It is a waxy, whitish, soft to solid material that forms from human soft tissues^20^. Following death, adipocere is produced after hydrolysis of triacylglycerols by intrinsic tissue lipases^20^. Adipocere is comprised mainly of the resulting saturated free fatty acids (C_16:0_ > C_18:0_ > C_14:0_), Na-, K-, Ca- and Mg-fatty acid soaps, hydroxy- and oxo-fatty acids, epicoprostanol and polyhydroxy fatty acids^20,22,23^. 10-Hydroxy stearic acid (10-OH-C_18:0_) is a typical constituent of adipocere and considered as product of enzymatic hydration of oleic acid^24^. Indeed, free fatty acids were the dominant compounds extracted from all adipocere samples (G1–4, I1, A1) (Table 2). Their relative abundance ranged between 93.4% (G1) and 99.9% (G3) of the summed steroids, ambrein and fatty acids (Table 2). These consistently high abundances clearly reflect the origin of fatty acids from adipose tissue and contrast with the minor, and variable, proportions of ambrein in the adipocere samples. Somewhat lower contents of solvent-soluble fatty acids in adipocere G1 and G2 may be due to higher proportions of fatty acid soaps that were not dissolved by dichloromethane. Major saturated fatty acids (C_16:0_ > C_18:0_ > C_14:0_ > C_18:1_) and the occurrence of 10-OH-C_18:0_ suggest extensive microbial conversion of the human tissues to adipocere in the grave environment. Unlike the adipocere, ambergris contained fatty acids (Table 2) in only minor concentrations (0.7%), with the major components being ambrein (93.9%), coprostanol and epicoprostanol (Table 2). Previous analyses of 43 jetsam ambergris samples showed an ambrein content of 81.1 ± 22.1%, with samples having much higher concentrations than adipocere^8^. Ambergris is often referred to as a coprolith, coprolite or fecalith^2,25^. Its origin from faecal concretions produced in the rectum of Sperm whales is reflected by abundant coprostanol, epicoprostanol and coprostanone^1,2,7,9^. Notably, all faecal steroids typical of fresh whale ambergris^3,7^, except coprostanone, were also detected in our adipocere samples (Fig. 2 and Table 2). While epicoprostanol and cholesterol have been previously reported as minor constituents^22^, this is to the best of our knowledge the first report of coprostanol, 5α-cholestanol and epicholestanol in human adipocere (Fig. 2 and Table 2). These steroids observed in adipocere, most likely originated from (individually or in combination): (i) original tissues (cholesterol)^22,26^, (ii) migration of faecal steroids into the adipocere (coprostanol)^27,28^; (iii) the microbial conversion of the pre-formed steroids in the predominately anaerobic grave environment (5α-cholestanol, epi-stanols)^29–31^.

## Ambrein Formation in Human Adipocere

Suzuki^32^ and Janistyn^33^, Beauregard^34^ and Gattefossé^35^ defined ambergris as an intestinal metabolic product. Whereas it is now generally accepted that ambergris is a coprolith^2^, it is still unknown, how and from which precursor molecules, ambrein is produced. A relationship to tissue and faecal steroids and triterpenes was discussed in the past^2^, as well as a partial microbial non-concerted cyclisation of squalene brought about through microbial action^2,3,17,18^ (squalene is also the precursor of cholesterol^36^). Although steroid analyses have been used as an indicator of faecal material in archaeology^29^, forensics^28,37,38^ and environmental pollution studies^30,39^, ambrein has, to date, not been reported as having any association with human faeces. The presence of advanced sterol transformation products such as epi-stanols, along with the considerable burial time of the human bodies studied herein (~40 years), points to advanced post-mortem microbial activity within the graves. Therefore, occurrences of ambrein probably result from long-term biotransformation processes after burial of the corpses, rather than the initial presence of ambrein in fresh human faeces, or tissues. Likewise, given that ambergris is produced in the intestines of sperm whales in marine settings, the origin of ambrein in human adipocere may more plausibly be linked to the post-mortem transformation of intestine contents than microbial activity in the adipocere itself.

## Environment of Ambrein Production

In burials, moist and anaerobic conditions promote adipocere formation^21^. When environmental conditions are constant, adipocere can endure as a stable degradation product over thousands of years^40^. The lack of fast fatty acid degradation pathways under anaerobic conditions fosters the preservation of fatty acids in a grave environment^23^. Ambergris and ambrein are formed in the intestinal tract of sperm whales, likewise under anaerobic conditions^2^. Whether ambrein is secreted from the whale itself or is produced by enteric bacteria is still unknown. Taken together, however, it is evident that the environments of ambergris and adipocere formation are similar in some respects. As both materials form over many years and mostly under anaerobic conditions, these conditions are obviously necessary for ambrein formation. Consequently, if microorganisms are involved in the production of ambrein, they probably prefer moist or wet, anaerobic environments.

Despite modern analogues synthesized in the laboratory, jetsam ambergris is the source of some of the most valuable odors and fixatives in perfumes. It is therefore surprising to find it in such an unexpected environmental context as human adipocere. This demonstrates a possible link to terrestrial ambrein production and it is proposed that ambrein might be also produced in faeces and faecal related material other than sperm whales and human adipocere. It is still not fully understood if ambrein can also be produced in fresh faecal matter, but due to its occurrences in the coprolith ambergris and adipocere, it is assumed that it is an indicator of aged faecal material. The association of ambrein formation to faecal material and ageing under moist oxygen-depleted environments certainly requires more attention in studies of other mammalian and geological samples. If ambrein is identified as a product of advanced microbial conversion of fresh faeces and faecal related material, it might be a useful indicator of aged faecal matter in future studies.

## Material and Methods

### Adipocere and ambergris material

**Figure 3:**
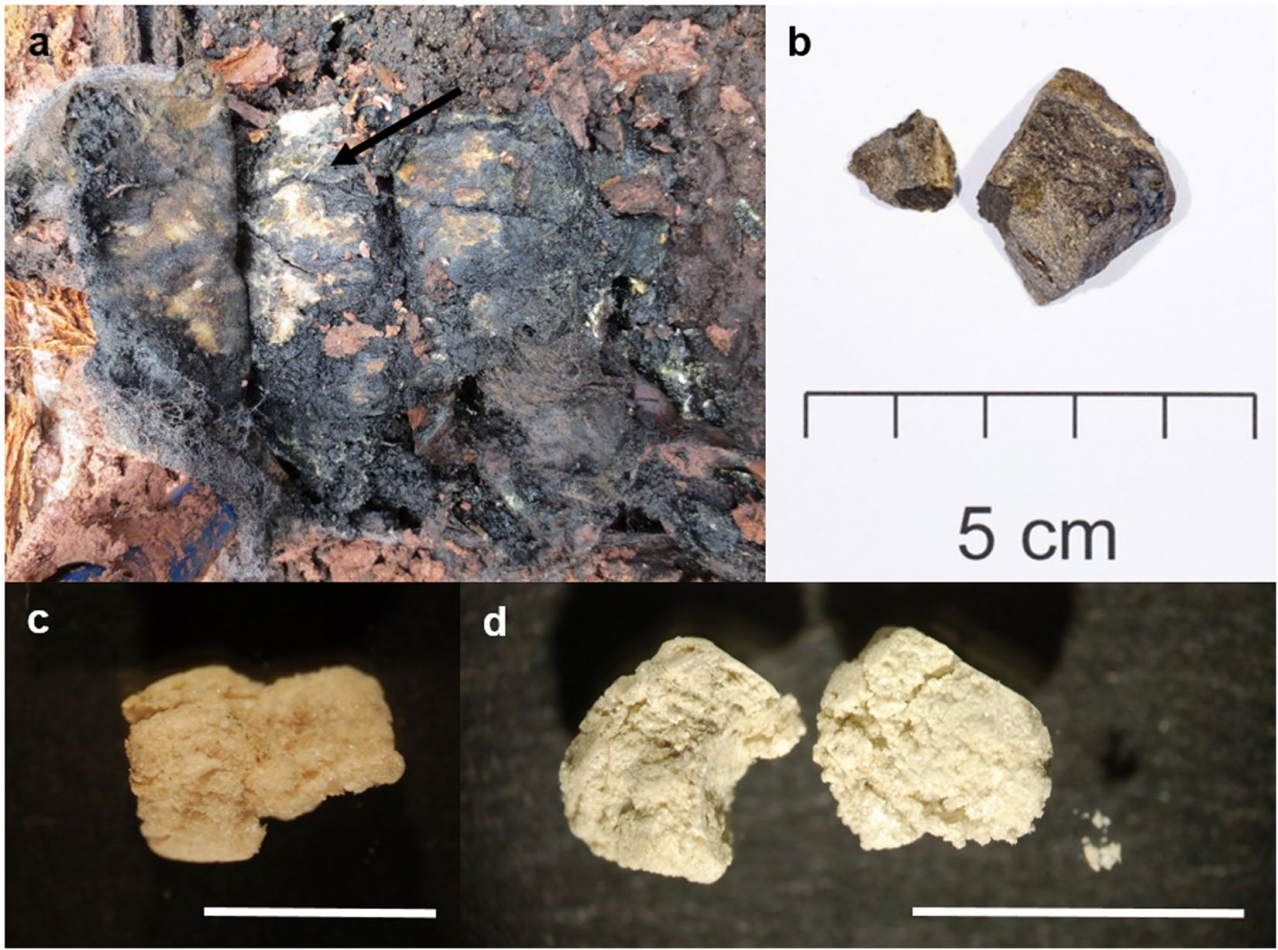
Photographs of adipocere and ambergris specimens. (**a**) Human corpse exhumation with adipocere; arrow points to adipocere formation on the human corpse, photo: M. Graw 2012. (**b**) Jetsam grey ambergris, Photo: H. Hadler 2014. (**c**) G3 adipocere sample (bar = 1 cm), photo: B. von der Lühe 2013. (**d**) G4 adipocere sample (bar = 1 cm), photo: B. von der Lühe 2013.

Research on the adipocere samples was approved by the ethics committee at the University of Göttingen. Applied methods were carried out in accordance with the relevant guidelines and regulations. Adipocere specimens (G1–4), obtained from graves, were sampled from exhumed bodies at a cemetery in the Black Forest, Germany (Fig. 3). Detailed descriptions of the adipocere formation at the cemetery are described by Fiedler *et al*. (2015) and Fiedler *et al*. (2012)^31,41,42^. Adipocere was obtained from the abdominal region of the corpses, I1 was obtained from the intestinal content of G2. The external adipocere specimen (A1) was kindly provided by Matthias Graw from the University of Munich. The adipocere samples were stored at −20 °C and were freeze-dried prior to analysis.

Jetsam ambergris was obtained from a private company in New Zealand specialising in providing ambergris samples for personal use. So-called ‘grey ambergris’ was analysed because it is known to contain high concentrations of ambrein and is of high quality for perfumery purposes^2^ (Fig. 3). The ambergris was kept in the dark at room temperature (21 °C).

### Neutral lipid extraction for ambrein and steroids

Subsamples of adipocere and ambergris were obtained with an acetone-rinsed spatula. For analysis, 2 mg of freeze-dried adipocere and ambergris were weighed in 4 mL glass vials. To remove fatty acids, direct saponification was carried out with 1.5 mL of 0.7 M methanolic KOH at 50 °C for 16 h. Neutral lipids were obtained with liquid-liquid extraction by adding 0.5 mL H_2_O and 2 × 1.5 mL *n-*heptane. The extracts were dried under nitrogen. Grave samples and the external adipocere (G1–4, I1, and A1) were directly subjected to derivatisation; the ambergris sample was diluted 1:100 v/v before derivatisation.

For silylation, 100 µL of *N*_,_*O*-Bis(trimethylsilyl)acetamide:chlorotrimethylsilane:1-(trimethyl-silyl)-imidazole (TMSIm/BSA/TMCS, 3/3/2, v/v/v; Sigma Aldrich, Taufkirchen, Germany) was added and extracts were heated at 90 °C for 1 h to form the trimethylsilyl (TMS-) derivatives of the compounds of interest. The samples were reconstituted in *n*-hexane containing 5α-cholestane as internal standard (IS; 5 mg L^−1^) and analysed by GC-MS.

### Fatty acid extraction

This extraction procedure extracts free dichloromethane soluble fatty acids in adipocere. Residues of the extracts are presumably fatty acid soaps, that were not further investigated. Subsamples of adipocere and ambergris were obtained with an acetone-rinsed spatula. For analyses, 2 mg of freeze-dried adipocere and ambergris were weighed in 10 mL glass tubes. Samples were subjected to sonication (3 × 5 min) in dichloromethane (4 mL) in order to extract the fatty acids. Dried aliquots of the extract were derivatised to methyl esters with trimethylchlorosilane (TMCS) in methanol (1:9, v/v) for 1 h at 80 °C. Aliquots were reconstituted in *n*-hexane containing methyl nonadecanoate (C_19:0_) as IS (4 mg L^−1^) and analysed by GC-MS.

### Instrumentation

Extracts were analysed using a Thermo Fisher Trace 1310 gas chromatograph coupled to a Thermo Fisher Quantum XLS Ultra mass spectrometer (ThermoFisher Scientific, Waltham, Massachusetts, USA). A ZB-5ms (Phenomenex, Torrance, USA), 30 m fused silica capillary column, 250 µm i.d. with a film thickness of 0.25 µm was used with helium (BIP^®^ ECD) as carrier gas at a constant flow of 1.5 mL min^−1^. The ambergris extract was injected in split mode 20:1; all other samples were injected splitless. The injection volume was 1 µL. The injection port was raised from 80 °C to 300 °C at 14.5 °C s^−1^. The column temperature was kept at 80 °C for 1 min, then increased to 310 °C at 5 °C min^−1^ where it was kept for 20 min. Solvent delay for filament activation of the ion source was set at 20 min and electron ionization voltage was set at 70 eV. The scanning mass range was *m/z* 50–600. Steroids and ambrein were identified according to their TMS-mass spectra published in the literature^3,19,29^, the NIST 05 Mass Spectral Search Program and by comparing mass spectra and elution order with steroid analytical standards (from Sigma Aldrich (Germany) and Chiron (Norway)) for coprostanol, epicholestanol, epicoprostanol, 5α-cholestanol and cholesterol.

## Supplementary information

Supplementary Material

## Data Availability

The authors declare that lists of the sources of published data used in this study are available within the article and its Supplementary Information Files.
