# Supplementary material for: First evidence of terrestrial ambrein formation in human adipocere
Source: Sci Rep. 2019 Dec 4;9:18370. doi: 10.1038/s41598-019-54730-w (PMC6892809; doi:10.1038/s41598-019-54730-w)
Supplement: Supplementary file 1 — Supplementary Material [file 41598_2019_54730_MOESM1_ESM.pdf]

SUPPLEMENTARY MATERIAL

First evidence of terrestrial ambrein formation in human adipocere

Barbara VON DER LÜHE[*1a], Robert W. MAYES[2], Volker THIEL[3], Lorna A. DAWSON[2], Matthias GRAW[4], Steven J. ROWLAND[5], Sabine FIEDLER[1]

[1]*Institute of Geography, University of Mainz, Johann-Joachim-Becher-Weg 21, 55099 Mainz, Germany*

[2]*The James Hutton Institute, Craigiebuckler, Aberdeen AB15 8QH, Scotland, UK*

[3]*Geobiology, Geoscience Centre, University of Göttingen, Goldschmidtstraße 3, 37077 Göttingen, Germany*

[4]*Institute of Forensic Medicine, University of Munich, Nußbaumstraße 26, 80336 Munich, Germany*

[5]*Petroleum and Environmental Geochemistry Group, Biogeochemistry Research Centre, University of Plymouth, Drake Circus, Plymouth, PLA 8 AA, UK*

[a]*now at: Physical Geography, Institute of Geography, University of Göttingen, Goldschmidtstraße 5, 37077 Göttingen, Germany*

*Corresponding Author:

Phone: +49 (0)551 394570

E-Mail: barbara.von-der-luehe@uni-goettingen.de

Material from main text

Figures and experiments referring to the main text

Mass spectra. In the following the electron impact mass spectra of TMS-ambrein is shown in in Fig. S1.

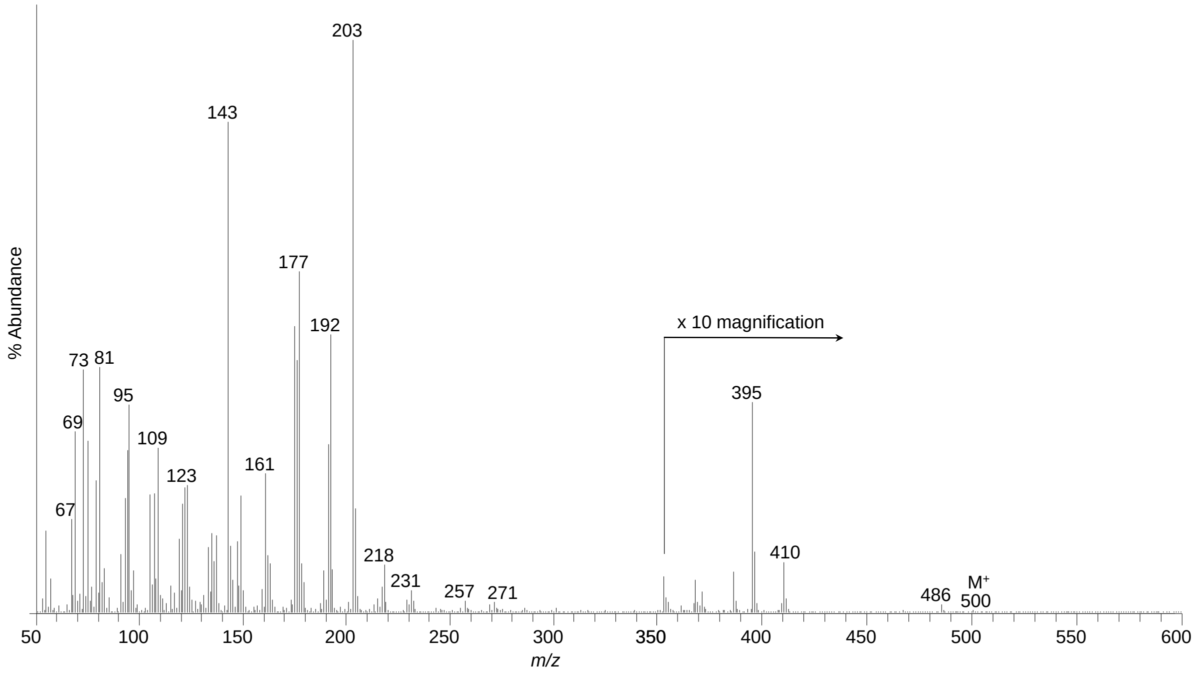

Figure S1: Electron impact mass spectrum of TMS-ambrein of adipocere sample G2.

Ambergris and adipocere co-elution. A derivatised ambergris extract was co-eluted with a derivatised adipocere extract (G2), to ensure similar retention times and mass spectrums of ambrein in both materials (Fig. S2). Subsamples of adipocere and ambergris were obtained with an acetone rinsed spatula. For analyses, 2 mg of freeze-dried adipocere and ambergris were weighed in 10 mL glass tubes. Samples were subjected to sonication (3 x 5 min) in dichloromethane (4 mL) in order to obtain the total lipid extract. Samples were dried under N_2 and aliquots with corresponding ambrein concentrations were silylated with 100 µL of *N,O*-Bis(trimethylsilyl)acetamide:chlorotrimethylsilane:1-(trimethyl-silyl)-imidazole (TMSIm/BSA/ TMCS, 3/3/2, v/v/v; Sigma Aldrich, Taufkirchen, Germany) at 90°C for 1 h. Aliquots were reconstituted in *n*-hexane and analysed with GC-MS according to the instrument set-up used in the Material and Methods chapter of the main text.

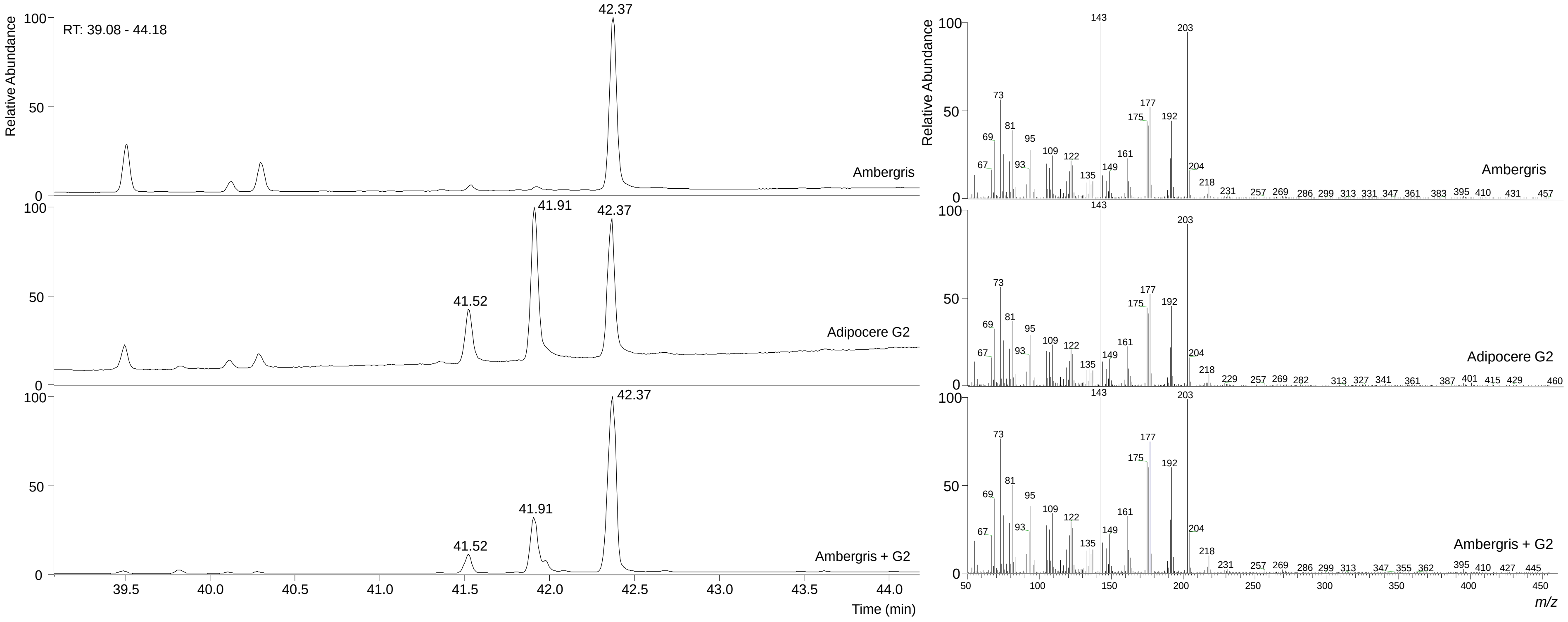

Figure S2: Co-elution experiment of a derivatised ambergris and a derivatised adipocere extract (G2). Partial (35 min to 47 min) total ion current GC-MS chromatograms with retention times of following compounds: 41.52 = coprostanol; 41.91 = epicoprostanol; 42.37 = ambrein and the corresponding electron impact mass spectrum of TMS-ambrein of the ambergris, adipocere (G2) and the co-eluted extract (ambergris + G2).

Preliminary work with GC-MS. Various test runs were conducted before publication of the results, to ensure proper analysis of ambrein in adipocere and ambergris with GC-MS. These test runs were conducted between 2013 and 2015 at the Institute of Geography at the Johannes Gutenberg University in Mainz, Germany and at the James Hutton Institute in Aberdeen, Scotland. Test runs are briefly summarised in the following:

To exclude the possibility that ambrein is formed during saponification, adipocere samples were extracted with DCM:acetone:glacial acetic acid (90:10:2 v/v/v) in an ultrasonic bath without saponification. Neutral lipids were separated from fatty acids by solid phase extraction (SPE). SPE-columns (3 mL volume) were fitted with PTFE-inserts and 1.5 mL silica gel (60 Å, 0.063-0.200 mm, in *n*-heptane; Merck, Darmstadt, Germany) and preconditioned with 2 x 1.5 mL n-heptane. Neutral steroids were eluted with 6 x 1 mL n-heptane:ethyl acetate (80:20, v/v). Extracts were derivatised with 50 µL *N,O*-Bis(trimethylsilyl)trifluoroacetamide+Trimethylchloro-silane (BSTFA+TMCS, 99:1, v/v) and pyridine (3:1, v/v) at 90°C for 1 h. The excess derivatisation agent was removed with nitrogen and samples were analysed in n-heptane by GC-MS (Agilent 6890N GC and 5975B MS; Agilent Technologies, Santa Clara, CA, USA) at the University of Mainz. GC-MS parameters and conditions are listed in Tab. S1. Ambrein was found in underivatised form (Fig. S3) in the sample extracts and was not produced during saponification, sample preparation and derivatisation. Consequently, the most commonly used silylation agent, BSTFA, was not used because of problems with incomplete derivatisation with this reagent. It was therefore decided to use the derivatisation method published by Rowland et al. 2018[2].

In a second test, an underivatised adipocere neutral lipid extract was run on GC-MS (Thermo Finnigan Trace GC/DSQ MS; Austin, TX, USA) at the James Hutton Institute for comparison with the mass spectrum of free ambrein published in the NIST library (Version 2.0). By subtraction of the background, a match of 95 % was achieved (90 % without subtraction) (Fig. S4). The characteristic mass ion at *m/z* 410 was attributed to the loss of water ($M^+ - H_2O$) of the molecular ion, which is typical for aliphatic alcohols[1].

Table S1: GC-MS parameters and conditions.

Parameter	Condition
**Column**	DB-5ms UI, 30 m x 0.25 mm x 0.25 µm
**Carrier gas**	He (99.9995%)
Flow	1.1 ml min^{-1}
**Injection**	
Volume	1 µl
Type	splitless mode
Temperature	250°C
**Oven**	
Initial temperature	80°C for 1.5 min
Rate	12°C min^{-1} to 265°C
	0.8°C min^{-1} to 280°C
	10°C min^{-1} to 300°C held for 12 min
**MS parameters**	
Acquisition mode	full scan
Scan parameters	50-600 m/z
Solvent delay	20 min
electron energy	70 eV

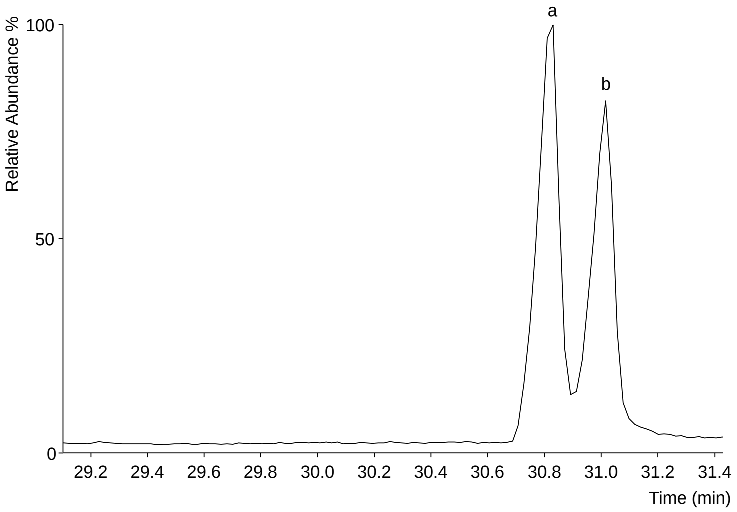

Figure S3: Partial total ion current GC-MS chromatograms of underivatised adipocere. Components a are underivatised coprostanol and epicoprostanol and b is underivatised ambrein.

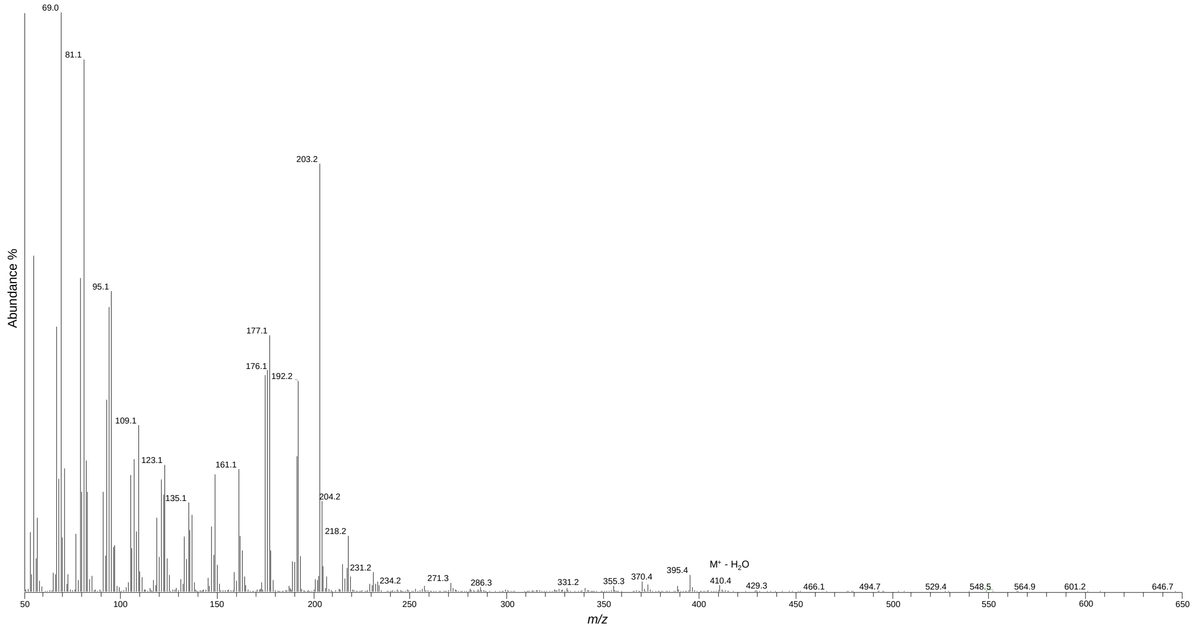

Figure S4: Electron impact mass spectrum of underivatised ambrein in adipocere. There was a 90% match of the NIST library mass spectrum of ambrein and 95% match with background subtraction. Mass spectrum of ambrein in NIST library by Governo et al. (1977)[1].

Mass spectra. In the following electron impact mass spectra of steroids in G1 and G2 adipocere are shown: TMS-coprostanol in Fig. S5, TMS-epicholestanol in Fig. S6, TMS-epicoprostanol in Fig. S7, TMS-cholesterol in Fig. S8 and TMS-5α-cholestanol in Fig. S9.

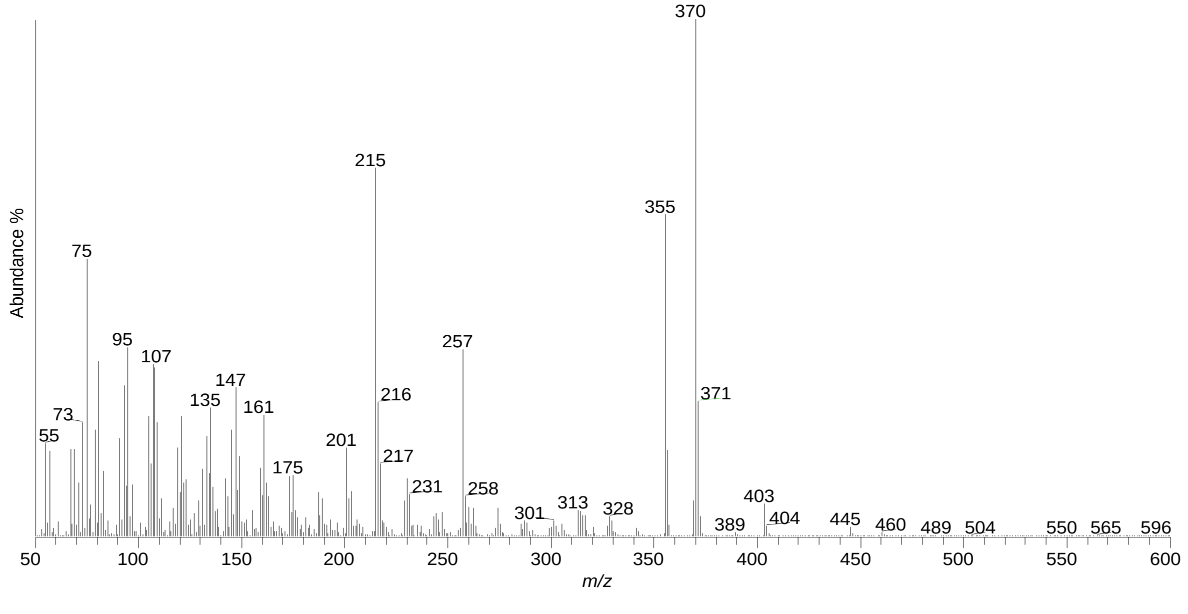

Figure S5: Electron impact mass spectrum of TMS-coprostanol of adipocere sample G1.

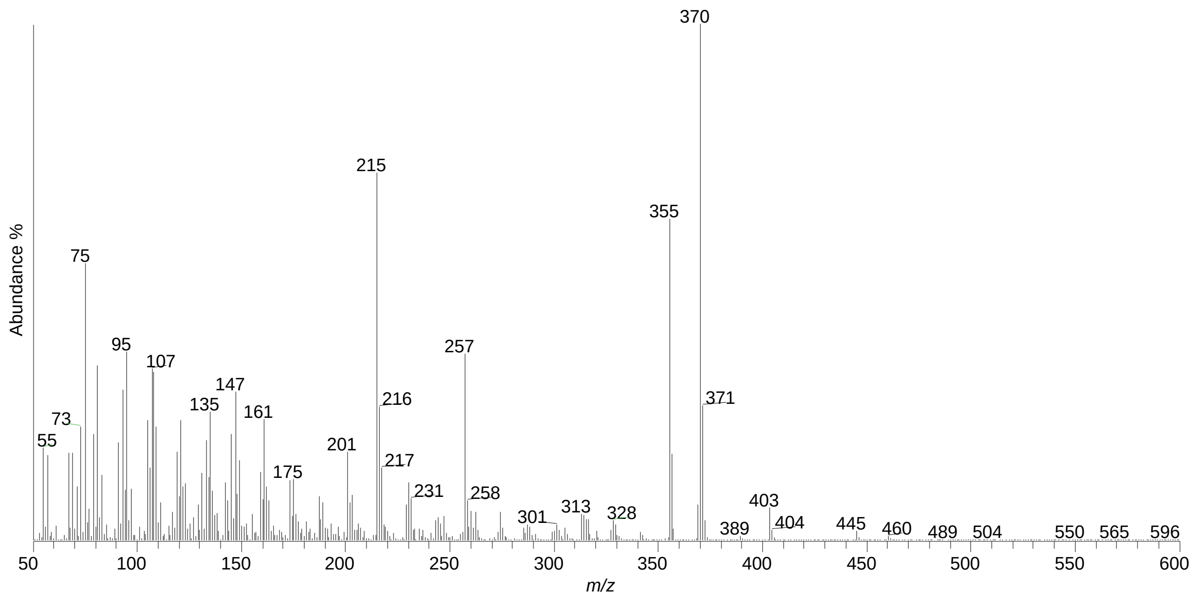

Figure S6: Electron impact mass spectrum of TMS-epicholestanol of adipocere sample G1.

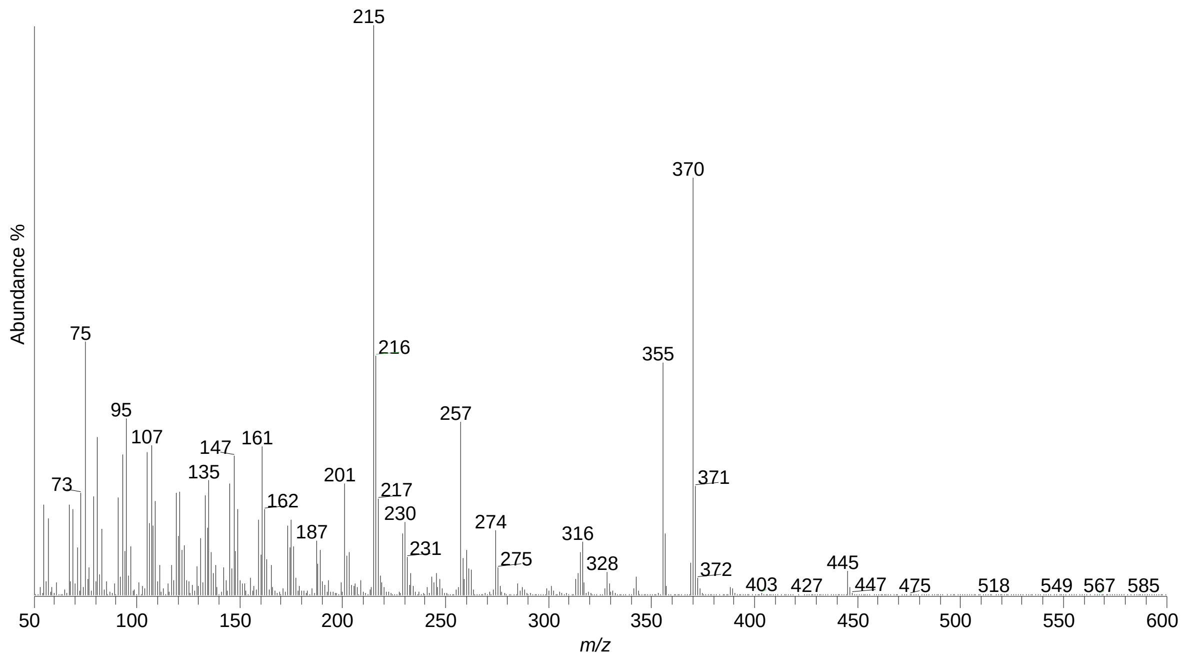

Figure S7: Electron impact mass spectrum of TMS-epicoprostanol of adipocere sample G1.

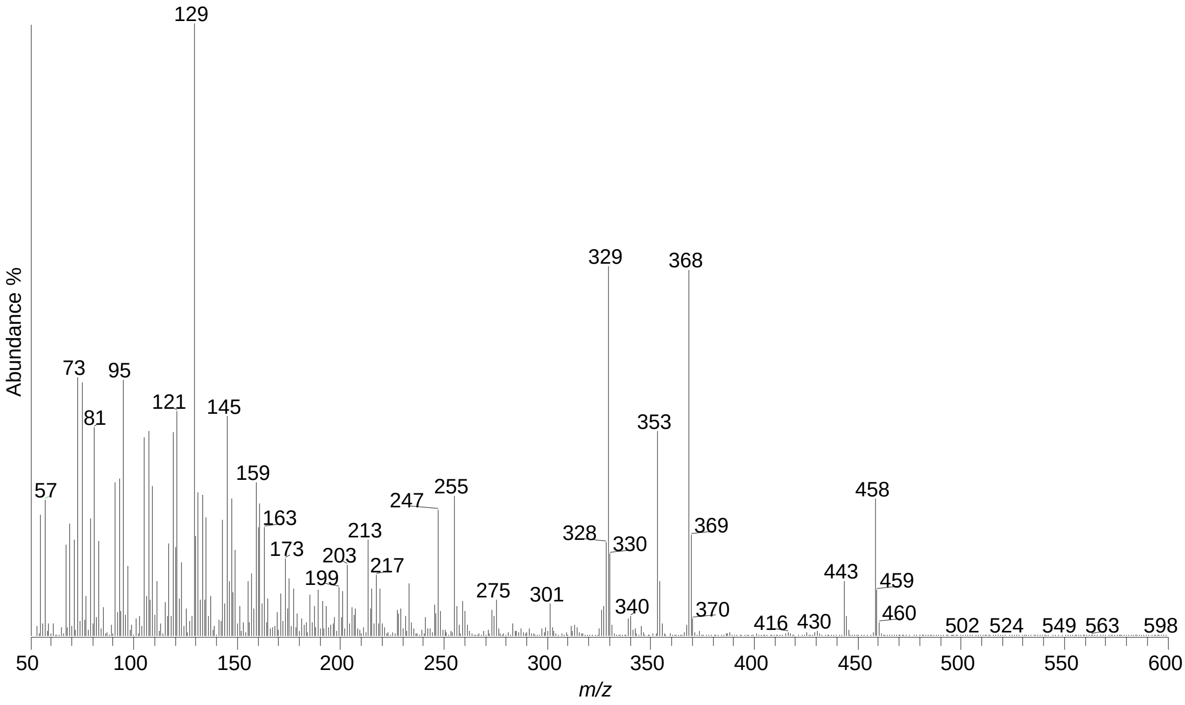

Figure S8: Electron impact mass spectrum of TMS-cholesterol of adipocere sample G1.

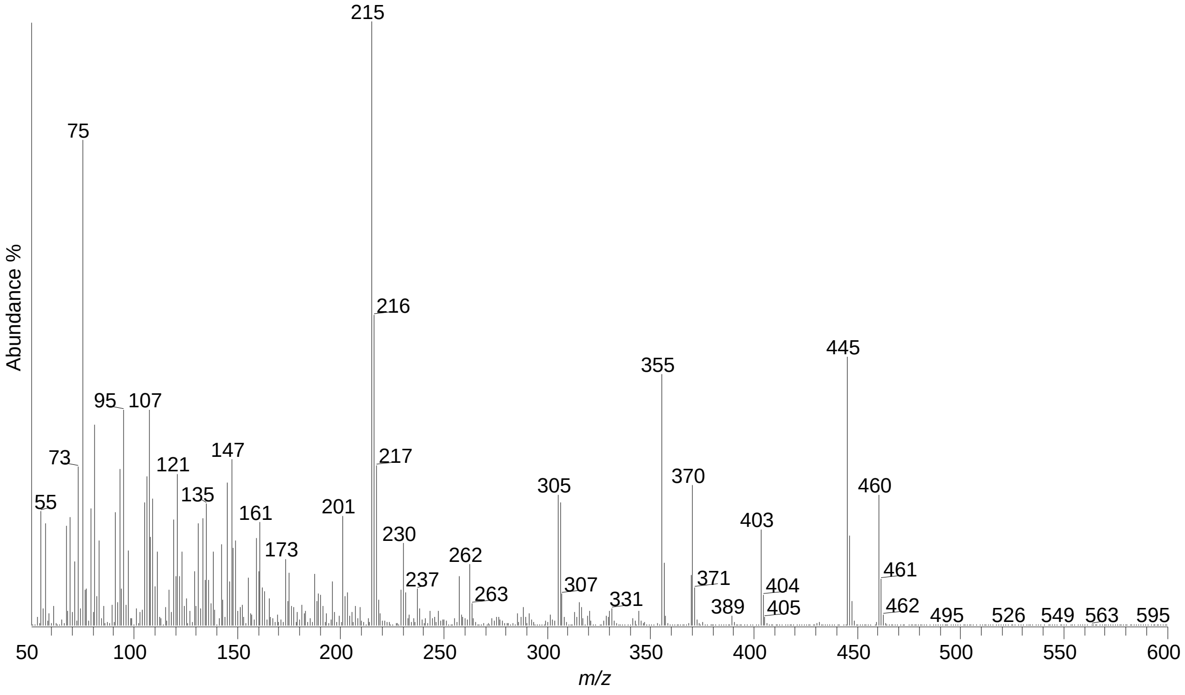

Figure S9: Electron impact mass spectrum of TMS-5α-cholestanol of adipocere sample G1.

References

1. Governo, T.F., Alessandro, R.T. & Prager, M.J. Gas-liquid chromatographic-mass spectrometric detection and identification of ambergris. J Assoc Off. Anul. Chem. Int. 60, 160–164 (1977).
2. Rowland, S.J., Sutton, P.A., Belt, S.T., Fitzsimmons-Thoss, V. & Scarlett, A.G. Further spectral and chromatographic studies of ambergris. *Nat. Prod. Res.* **22**, 1–7 (2018).
